# The Role of the Z-DNA Binding Domain in Innate Immunity and Stress Granules

**DOI:** 10.3389/fimmu.2020.625504

**Published:** 2021-02-03

**Authors:** De Chen Chiang, Yan Li, Siew Kit Ng

**Affiliations:** ^1^ Advanced Medical and Dental Institute, Universiti Sains Malaysia, Kepala Batas, Malaysia; ^2^ School of Pharmaceutical Sciences, Universiti Sains Malaysia, Gelugor, Malaysia; ^3^ Department of Biology, Southern University of Science and Technology, Shenzhen, China

**Keywords:** Z-DNA binding domain, Z-RNA, innate immunity, stress granules, ADAR1, ZBP1, E3L, PKZ

## Abstract

Both DNA and RNA can maintain left-handed double helical Z-conformation under physiological condition, but only when stabilized by Z-DNA binding domain (ZDBD). After initial discovery in RNA editing enzyme ADAR1, ZDBD has also been described in pathogen-sensing proteins ZBP1 and PKZ in host, as well as virulence proteins E3L and ORF112 in viruses. The host-virus antagonism immediately highlights the importance of ZDBD in antiviral innate immunity. Furthermore, Z-RNA binding has been shown to be responsible for the localization of these ZDBD-containing proteins to cytoplasmic stress granules that play central role in coordinating cellular response to stresses. This review sought to consolidate current understanding of Z-RNA sensing in innate immunity and implore possible roles of Z-RNA binding within cytoplasmic stress granules.

## Introduction

### Z-DNA/Z-RNA and Zα Domain

The structure of double-stranded DNA (dsDNA) in nature can be broadly categorized into 3 major forms, namely compact right-handed A-DNA, loose right-handed B-DNA and the unique left-handed Z-DNA conformation. Unlike the anti-conformation base arrangement throughout A- and B-DNA, the nucleoside bases in Z-DNA adopt alternating syn- and anti-conformation bases, giving rise to its distinctive left-handed double helical structure with zigzag backbone (thus its name) (1). The X-ray crystal structure of dsDNA in Z-conformation was first observed in the 1980s under high salt condition (15mM MgCl_2_) (2, 3). However, it is only after the discovery of the first Z-DNA binding domain (ZDBD) in Adenosine Deaminase Acting on RNA 1 (ADAR1) protein almost two decades later that implied physiological-relevance of Z-DNA (4). Since then, innumerable studies were done to identify ZDBD in other proteins, and characterize the role of these ZDBD-containing proteins in gene regulation, innate immunity, cancer, and autoimmunity disease (5–8). Similar to dsDNA, double-stranded RNA (dsRNA) can also adopts the Z-conformation (9). Indeed, both Z-RNA and Z-DNA can be stabilized by the first ZDBD of ADAR1, where resolved crystal structure revealed nearly identical protein-nucleic acid interactions for both Z-RNA and Z-DNA to form thermodynamically stable complexes (10–12). Therefore, it is widely assumed that Z-RNA interacts with ZDBDs in a similar manner to Z-DNA.

Under normal physiological conditions, Z-DNA/Z-RNA exist at higher energy configuration and thus are energetically unstable on their own, except when in binding with ZDBDs (13). The highly conserved ZDBD likely arise from a sub-group of winged Helix-Turn-Helix (wHTH) motif common in most prokaryotic DNA-binding proteins (13). In alignment with review article by Rich and Zhang, 2003 (1), ZDBDs that can form quasi-stable interaction with Z-DNA/Z-RNA will be referred to as the Zα domain, whereas homologous ZDBD that cannot will be mentioned as the Zβ domain, within this review. The Zα domain is highly-conserved, where the amino acid residues within the binding pocket primarily interacts with the zigzag sugar-phosphate backbone of Z-DNA/Z-RNA, which contributes to high specificity and affinity for Z-DNA/Z-RNA (10, 13, 14). Structural analysis revealed the binding preference of Zα domain to CG-repeat sequences, d(CG)_n_ (15). That said, some non-CG-repeat with similar structural features, d(CACGTG)_2_, d(CGTACG)_2_ and d(CGGCCG)_2_, have also been reported to bind to Zα domain (16). Numerous attempts to map Z-DNA formation in the nucleus had suggest its involvement in transcriptional regulation, but similar mapping data about Z-RNA is lacking (17, 18).

Since the initial discovery of Zα domain in ADAR1, Z-DNA/Z-RNA binding has been thought to be involved in mediating innate immunity, as Zα domain is only present in the interferon-inducible ADAR1_p150_ isoform, instead of the constitutively expressed ADAR1_p110_ isoform (19, 20). In addition to mammalian ADAR1, Zα domains were subsequently discovered to be encoded in proteins across different classes of metazoans, including immunity-related Z-DNA Binding Protein 1 (ZBP1) in mammals (21) and Protein Kinase Containing Z-DNA binding domains (PKZ) in fishes (22); and viruses affecting them, such as E3L in poxviruses (23), and ORF112 in fish herpesviruses (24). RBP7910 is recently discovered in kinetoplastids as another ZDBD-containing protein (25). The recurring theme of Zα domains conservation within host immune proteins and pathogen proteins is highly indicative of the involvement of Zα domains in host immune regulation, against viral infections and/or in cancer and auto-immunity.

In this review, we focused on the current understanding for the role of Zα-containing proteins in innate immunity and post-transcriptional regulations, through their interaction with cytosolic Z-RNA.

### Antiviral Innate Immunity

Antiviral innate immunity generally refers to the initial programmed broad-spectrum cellular reaction following identification of viruses or their components. In a nutshell, the acute antiviral response starts with the sensing of virus-associated molecular patterns (VAMPS), followed by the cascading signaling events culminating in the activation of type I interferon response. Various host sensor proteins such as Toll-like receptors (TLRs), retinoic acid inducible gene- I (RIG-I)-like receptors (RLR), and Nod-like receptors (NLR) can recognize a vast range of viral nucleic acids or other VAMPS upon virus infection (26–28). These interactions kickstart the IRF3/7-, NF-κB-mediated signalling cascades towards the establishment of antiviral state in the infected and surrounding cells. The synthesis and secretion of type I interferons (IFN), IFN-α and IFN-ß, are of central importance in acute antiviral response (29, 30). Type I IFNs raise alarm in neighboring cells when bound to their IFN-α/ß receptors (IFNARs) (31), which in turn activate the Janus kinase (JAK)-Signal transducer activator of transcription (STAT) pathway (32). Phosphorylated STAT1 and STAT2, together with IRF9 (33), forms the Interferon Stimulatory Gene Factor 3 (ISGF3), a potent transcriptional activator of hundreds of interferon-stimulated genes (ISGs) (34, 35). The ISG protein effectors directly target viral functions and pathways to inhibit viral entry, translation, replication and egress; or promote intercellular communication to enhance pathogen sensing; or facilitate the resolve to cellular homeostasis during post-infection (36, 37). Persistent virus infection beyond the acute phase would lead to activation of virus-specific adaptive immune response, through IFNs and other pro-inflammatory cytokines (38).

### Stress Granules

Cellular stress occurs when the ability to maintain homeostasis balance within a cell is affected. Various factors including viral invasion, heat shock, oxidative stress, nutrient deprivation, DNA damage, can trigger stress response, where cell survival is dependent on successful resolution of the cellular stresses (39). Upon stress onset, cells will rapidly arrest their translational machinery and stall their protein synthesis (40). These arrested messenger ribonucleoproteins (mRNPs) will aggregate into multiple non-membranous foci in the cytoplasm, known as stress granules (SGs). The composition of SGs consists of ribosomal components, mRNA, structural proteins, and many signaling proteins (41). In addition, nucleocytoplasmic transport is also disrupted through recruitment of essential nucleocytoplasmic transport factors, including Ran GTPase, nucleoporins and karyopherins to SGs (42).

While initially assumed to be passive repositories of untranslated mRNA, SGs are now thought as RNA triage sites where mRNA transcripts were actively sorted towards decay, storage, or translation reinitiation (43). SGs function as vital signaling hubs in coordinating cellular processes during stress response, from selective translation of vital proteins against stress conditions, moderating metabolism, suppressing apoptosis, to antiviral response (44–46). Attempts to catalogue protein components within mammalian SGs suggests that many signaling and regulatory proteins moved in and out of SGs in spatiotemporal manner (47, 48). That said, there are some notable SGs markers including T-cell intracellular antigen 1 (TIA-1), TIA-1-related protein (TIAR), Ras GTPase-activating protein-binding protein 1 (G3BP1) and poly(A)-binding protein 1 (PABP1).Upon resolution or sufficient adaptation to the stress, step-wise dissolution of SGs allows stored mRNPs to quickly reform the translational assembly, therefore facilitating rapid recovery of protein translation in the cells (49).

The phosphorylation of translation initiation factor (eIF2α) by eIF2α kinases catalyses the formation of SGs through stalling of the initiation of ribosomal translation. The recycling of inactive eIF2α-GDP to active eIF2α-GTP is inhibited by eIF2α phosphorylation, thereby disrupting the formation of the essential translation initiator, $eIF2\alpha -GTP-tRNA_{i}^{Met}$ (50). The eIF2α kinases are activated under different stress conditions, for example PKR is activated by dsRNA, PERK by endoplasmic reticulum stress, while HRI and GCN2 is responsible for oxidative and nutrient stress respectively (51).

A recent excellent review has highlighted the role of SGs in antiviral response, notably on the mechanisms of viral translational inhibition and counteracting strategies adapted by viruses (46).SGs formation effectively arrests viral replication by sequestering viral mRNA into SGs from protein translation (27, 52). Intriguingly, while several viruses evolved various mechanism to inhibit SGs formation, such as promoting cleavage of G3BP1 (53), inhibiting PKR phosphorylation (54), or sequestering SGs core proteins (55, 56); others hijack the SGs formation to prioritize viral protein synthesis (57, 58).

## ZDBD-Containing Proteins

### Adenosine Deaminase Acting on RNA 1 (ADAR1)

**Figure 1:**
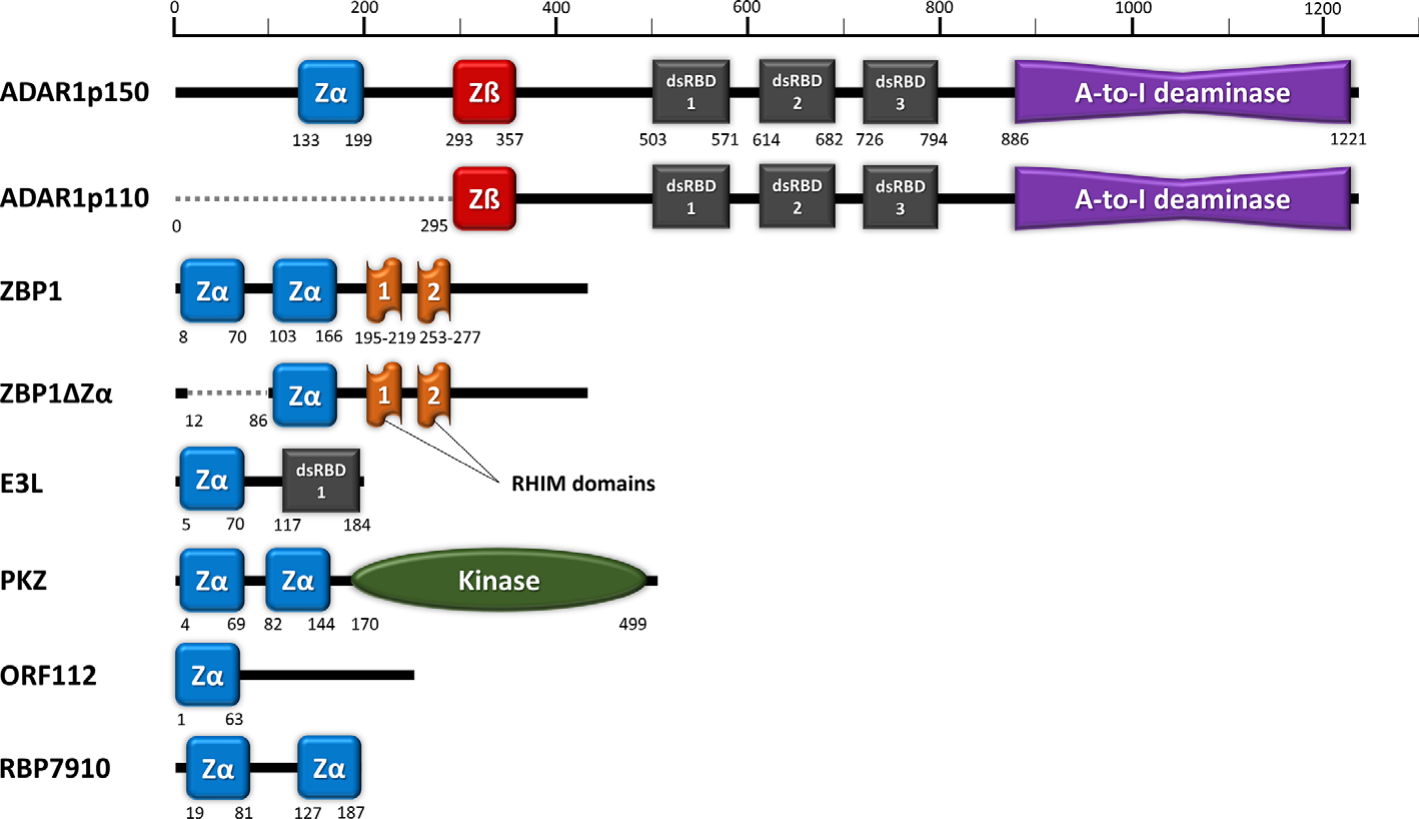
Domain organization of ZDBD-containing proteins: ADAR1, ZBP1, E3L, PKZ, ORF112, and RBP7910. Zα domain (blue) denotes ZDBD that can bind Z-DNA/Z-RNA, while Zß domain (red) denotes ZDBD that cannot bind Z-DNA/Z-RNA. ADAR1_p150_ harbors an extra Zα domain compared to ADAR1_p110_, while sharing identical dsRNA binding domains (dsRBDs) and catalytic deaminase domain. Two natural isoforms were also described for ZBP1, where ZBP1ZΔ does not contain the first Zα domain present in full length ZBP1. Vaccinia virus E3L vital for its pathogenicity contains a single Zα domain and a dsRBD. Fish PKZ contains two N-terminal Zα domains as the RNA recognition motifs, in addition to a C-terminal kinase domain. On the other hand, the ORF112 protein identified from fish herpesvirus only has a single Zα domain at its amino-end. Most recently, trypanosome RBP7910 protein has been reported to contain two Zα-like domains, although further characterization of their function may be necessary.

ADAR1 is a member of the Adenosine deaminase, RNA-specific (ADAR) protein family, where their adenosine-to-inosine (A-to-I) RNA editing activity is responsible for a wide range of regulation in gene expression, peptide modification in nervous system, RNA interference (RNAi), protein activation or inhibition (59). A-to-I dsRNA editing alters stable canonical U-A base pairing to U-I wobble, destabilizing the edited target dsRNA duplex conformation and compromising its functionality (60). In addition, ADAR1-mediated RNA editing activities are also responsible for a subset of cancer and tumour development (61), for instance, in gastric (62), cervical (63), breast (64), thyroid (65), liver (66) and colorectal cancers (67). The general structures of ADAR proteins consist of a deaminase domain at the C-terminal, and dsRNA binding domains (dsRBD). In addition, the human ADAR1 has extra N-terminal ZDBDs, a unique feature otherwise absents in ADAR2 and ADAR3 (**Figure 1**). There are two major ADAR1 isoforms; where ADAR1_p150_ is only induced by type I interferons and is mostly cytoplasmic, whereas the constitutively expressed ADAR1_p110_ is localized in the nucleus (68, 69). This distinct localization of ADAR1 isoforms is attributed to the presence of a bipartite Nuclear Export Signal (NES) located within the ADAR1_p150_-exclusive Zα domain, while the Nuclear Localization Signal (NLS) is within the common dsRBD region (70).

**Figure 2:**
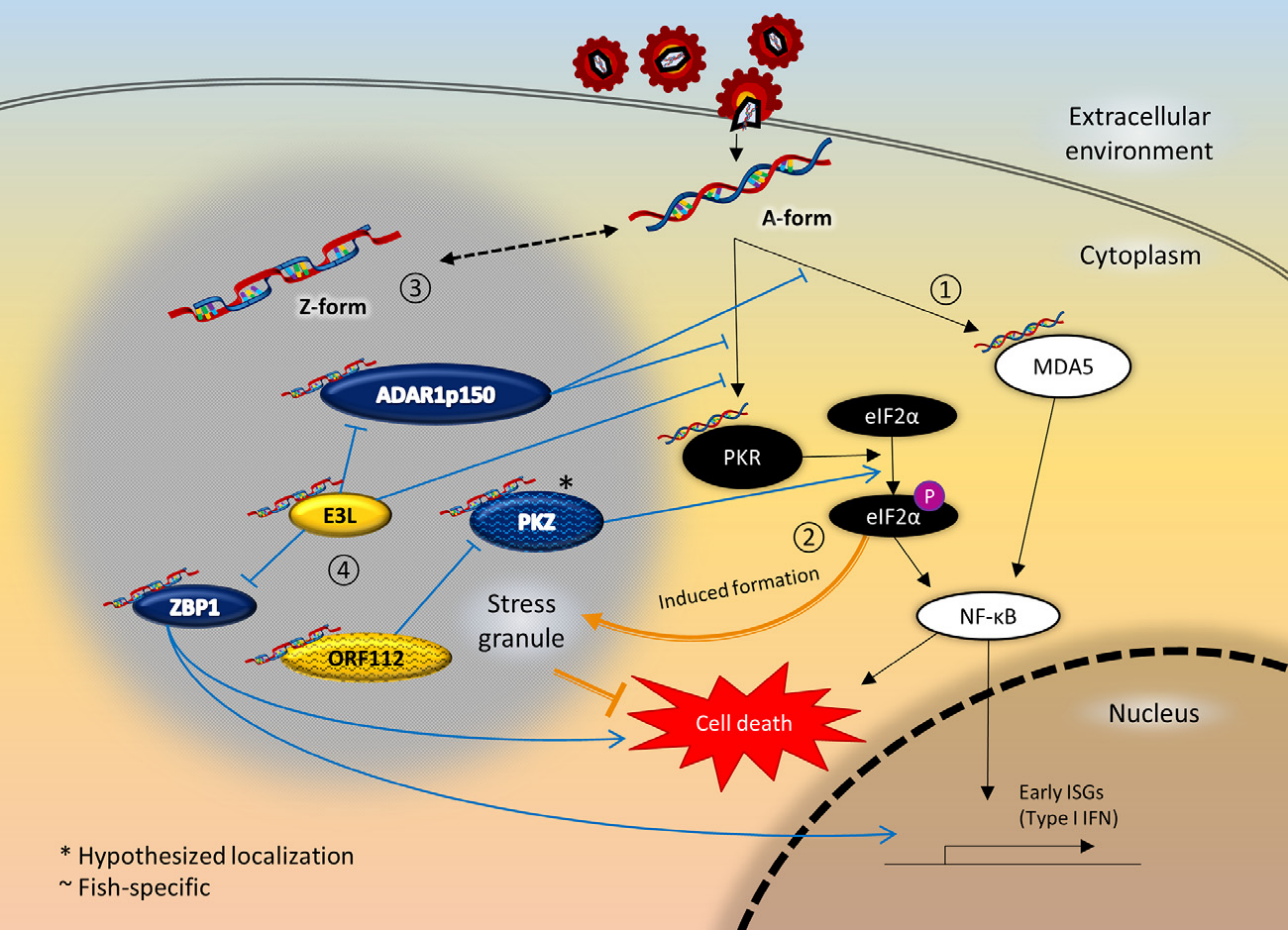
Crosstalk between ZDBD-containing proteins, stress granules, and innate immunity signaling pathway following RNA virus infection. 1) Innate immune sensors like MDA5 and PKR are activated upon recognition of virus RNA in the cytoplasm, which in turn activates the NF-κB pathway culminating in expression of type I IFN. 2) At the same time, activated PKR also phosphorylates eIF2α, causing stalled mRNA translation that induce the formation of stress granules. Stress granules are instrumental in promoting cell survival under cellular stresses like virus infection. 3) Most ZDBD-containing proteins have been shown to localize to stress granules, mediated *via* respective Zα domains. Therefore, it is possible that RNA can adopt Z-conformation more readily within stress granules. 4) ADAR1_p150_ is a negative regulator of immune response protecting against autoimmunity and chronic inflammation, as edited RNA transcripts lose immunogenicity. On the other hand, ZBP1 activation triggers IFN response and cell death mechanism. The vaccinia virus E3L is an inhibitory protein towards the antiviral response of its mammalian host, which is dependent of its Zα domain. In fish, PKZ is a paralog that complements PKR-mediated pathways, as PKZ can be activated by Z-RNA binding. The cyprinid herpesvirus ORF112 protein antagonizes the activation of PKZ as the virus immune evasion strategy. Incidentally, PKR and MDA5 had also been reported to localize to stress granules. It is therefore plausible that the close proximity through localization to stress granules facilitate the crosstalk between ZDBD-containing proteins and immune recognition and signaling pathways.

ADAR1 has been implicated as a master regulator of the innate immunity, largely through its A-to-I editing activity to avoid unwarranted deleterious effects (71–73). ADAR1 knockout studies showcased the vital regulatory role of ADAR1_p150_ in antiviral immune homeostasis and autoimmunity, through MDA5-MAVS sensing pathway (74), NF-κB gene regulation pathway (75) and PKR-mediated apoptosis (76) (**Figure 2**). Editing on ubiquitous self RNA such as Alu transcripts prevents recognition by cytosolic dsRNA sensor MDA5 and erroneous autoimmune response (77, 78). Meanwhile, A-to-I editing can disrupt both the viral translational and replication process by compromising the structural integrity and genetic consistency of virus RNA (79). Direct suppressive effect on viral replication by ADAR1 were observed in hepatitis C (HCV) (80) and hepatitis B (HBV) (81). On the other hand, numerous viruses have evolved to hijack ADAR1’s editing activity to as immune evasion strategy, since A-to-I editing of their RNA can avoid the innate immune sensing (82). The immune suppressive ability of ADAR1 were exploited by measles virus (83), hepatitis delta virus (HDV) (84), Human Immunodeficiency Virus (HIV) (85) and Kaposi’s sarcoma-associated herpesvirus (KSHV) (86). For Human T-cell leukaemia virus type 1 (HTLV-1) (87) and dengue virus (DENV) (88), only overexpression of the cytoplasmic ADAR1_p150_, but not ADAR1_p110_, had pro-viral effect.

The exact role of the exclusive Zα_ADAR1_ in ADAR1_p150_ has only been slowly unravelled in recent years. Rare Mendelian autoimmune disorders like Aicardi-Goutières syndrome (AGS) and Bilateral Striatal Necrosis/Dystonia (BSD) have been attributed to ADAR1 dysfunction. Genetic profiling of *Adar1* gene among these patients showed that loss-of-function mutation at Zα_ADAR1_ causes the hallmark type I interferonopathy, suggesting that Z-DNA/Z-RNA binding is important to prevent dysregulated interferon response (89). In addition, Zα_ADAR1_ is found to be responsible for localization of ADAR1_p150_ to SGs under oxidative and interferon-induced stress (90, 91) (**Figure 2**). In contrast, ADAR1_p110_ lacking the Zα_ADAR1_ does not localize to SGs (91) (**Figure 1**). Only mutation to key interacting residues at the binding pocket of Zα_ADAR1_ affects the localization to SGs, showing that Z-RNA binding is indeed important (91). Z-RNA binding ability is essential in Zα_ADAR1_-ribosome interaction, which leads to translational inhibition (92). On the other hand, ADAR1-kd cells exhibit an increase in SGs formation following virus infection or IFN-treatment, suggesting inhibitory role of ADAR1 on SGs formation (93).This is in line with the general observation whereby ADAR1 functions as a suppressor of type I interferon response, including inhibiting the PKR phosphorylation precluding SGs formation (77). Enhanced editing ability has been described on dsRNAs that more readily adopt Z-conformation, where Z-RNA binding could alter the selectivity of ADAR1-editing site and dsRNA substrates (94).

### Z-DNA-Binding Protein 1 (ZBP1)

Z-DNA-binding protein 1 (ZBP1), alternatively known as DNA-dependent activator of IFN-regulatory factors (DAI) or Tumor stroma and activated macrophage protein (DLM1), plays a significant role in innate immune response against viruses or other non–self-agents (95). ZBP1 has two conserved N-terminal Zα domains, and two receptor-interacting protein homotypic interaction motif (RHIM) domains (**Figure 1**). The presence of only Zα domains as nucleic-acid recognition domains suggests the primary role of ZBP1 as a cytosolic sensor for Z-DNA/Z-RNA. Activation of ZBP1 then leads to downstream signal transduction mediated through the RHIM domain interactions with receptor-interacting protein (RIP) kinases, regulating apoptosis, inflammation, and interferon responses to pathogens (95, 96). In addition, ZBP1 sensing activates NLRP3 inflammasome complex that leads to PAN-optosis (pyroptosis, apoptosis, and necroptosis) process (97) (**Figure 2**). In mice model, knocking out ZBP1 is sufficient to ameliorate IFN-γ- and TNF-induced acute systemic inflammatory response syndrome (SIRS), which demonstrates its involvement in IFN-induced necroptosis (98). ZBP1-knockout mice exhibited remarkably reduced inflammatory responses and epithelial damage than the wild type mice upon influenza A virus infection, suggesting the essential role of ZBP1 in PAN-optosis pathways (99). ZBP1 deletion significantly increased the mortality rate in mice when infected with West Nile virus (WNV) and Zika virus (ZIKV) (100). ZBP1 has also been implicated IL-17-mediated skin inflammation (101) and regulation of stem cell differentiation (102).

X-ray crystallographic study has revealed significant Z-RNA/Z-DNA binding affinity for both first and second Zα_ZBP1_ (103). Somewhat confusingly, only the first Zα_ZBP1_ is essential for the localization of ZBP1 to cytoplasmic SGs under heat, arsenite and interferon-induced stress (91, 104). Meanwhile, a prominent alternatively-spliced ZBP1 variant without the first Zα domain (ZBP1ΔZα1) does not localize to SGs (104) (**Figure 1**). Interestingly, the ZBP1ΔZα1 variant forms aggregates in untreated cells that disassemble upon heat shock or arsenite treatment, in a total contrast to full length ZBP1. The Z-RNA generated from anti-sense Influenza A virus (IAV) triggers RIPK3-MLKL-mediated necroptosis, where nuclear envelope collapse in infected cells leads to cell death and neutrophil recruitment (105). In another study, the second Zα_ZBP1_ is found to be essential for influenza-induced PAN-optosis (106). For Herpes simplex virus (a DNA virus), ZBP1-mediated necroptosis is thought to be activated through interaction with viral RNA transcripts, instead of viral DNA (107).

### Protein E3 (E3L) of Poxvirus

Intriguingly, the poxvirus E3L protein reportedly vital for the virulence and host range factor, viral pathogenesis, and antagonizing host innate immunity, also contains Zα domain (23). The E3L protein suppresses cytokines-mediated inflammation through both PKR-dependent and PKR-independent pathways; in which p38 and NF-κB activation is inhibited, and IL-6 and IFN-ß production ameliorated, respectively (108). The 184 amino acid long E3L protein harbours two distinct RNA binding motifs, a conserved Zα domain at N-terminal and a dsRBD at the C-terminal (**Figure 1**). Both domains work synergistically to suppress immune response to poxvirus infection, although the mechanism is not entirely clear. The phosphorylation of antiviral transcription factors IRF3 and IRF7 can be effectively inhibited by E3L without PKR-dsRNA interactions (109). Interestingly, mutational analysis revealed that dsRNA binding activity is not necessary for antagonistic role of E3L protein for PKR inhibition, cytokine suppression and apoptosis (110). Antiviral factor ISG15 inhibition requires the dsRBD to block type I interferon (IFN) induction (111), however, Zα_E3L_ is imperative for full IFN and PKR inhibition (112, 113).

Zα_E3L_ exhibits high structural resemblance to those of ADAR1 and ZBP1, and is capable of Z-DNA/Z-RNA binding. Indeed, the Zα_ADAR1_ and Zα_ZBP1_ can functionally replace Zα_E3L_ without affecting the viral pathogenicity of E3L protein (114). Therefore, Zα_E3L_ likely plays a role in competitive inhibition for Z-DNA/Z-RNA binding antagonizing the function of ADAR1_p150_ and ZBP1 during virus infection (114). The Zα_E3L_ is responsible for suppressive effects in toll-like receptor (TLR) activation and host immune response against vaccinia virus infection (115). A recent study directly demonstrated Zα_E3L_ as a competitive inhibitor with ZBP1, whereby the masking of putative Z-DNA/Z-RNA prevents RIPK3-mediated necroptosis (116) (**Figure 2**).

Consistent with ADAR1_p150_ and ZBP1, the Zα_E3L_ is also responsible for its localization to mammalian SGs, mediated *via* functional Z-RNA binding (91) (**Figure 2**). Indeed, vaccinia virus mutant lacking E3L causes elevated SGs assembly, translational arrest, and reduced viral replication within the mammalian cells (117).

### Protein Kinase Containing Z-DNA Binding Domains (PKZ)

PKZ is an immune modulator protein initially discovered in fishes as a paralog to PKR (118–120). PKZ shares a similar C-terminal catalytic domain with PKR, but differs at the N-terminal RNA binding region. PKZ harbours two Zα domains at its N-terminal, instead of two dsRBDs in PKR (**Figure 1**). Although both PKR and PKZ have independent sensing mechanism for dsRNA and Z-form nucleic acids, these proteins demonstrate a cooperative role in host response against viral infection (121). The conservation of PKZ among fishes suggests expanding the pathogen- or danger-associated molecular patterns (PAMPs/DAMPs) recognition is important for fish immunity and survival (122). Similarly to PKR, PKZ can initiate apoptosis *via* eIF2α phosphorylation in viral-infected cells as part of its antiviral role (8) (**Figure 2**). Significant interactions were observed through coimmunoprecipitation assays between cytosolic PKZ with other IFN immune mediators like IRF3, IRF9 and STAT2, illustrates the vital role of PKZ in inducing fish’s IFN response (123). A recent review details the role of PKZ within the type I interferon response of fish innate antiviral immunity (118).

Functional analysis revealed PKZ can only be activated by Z-DNA/Z-RNA binding, instead of poly(I:C)—a common viral dsRNA mimic (124) (**Figure 2**). Circular dichroism spectroscopy of the Zα_PKZ_ reported similar Z-DNA-binding affinity and facilitate efficient B-to-Z transition of bound nucleic acid ligand, in close correspondence to those interferon-inducible mammalian ZDBD-containing proteins described above (119, 125–127).

### Open Reading Frame 112 Protein (ORF112) of *Cyprinid herpesvirus 3*

The ORF112 protein of *Cyprinid herpesvirus 3 (CyHV3)*, a major koi herpesvirus infecting common carp, contains an N-terminal Zα domain (128) (**Figure 1**). ORF 112 of *CyHV3* is important in suppressing type I interferon response in infected teleost fishes, in comparison to spring viremia when infected with *Rhabdovirus SVCV* (129). This discovery of ZDBD in fish viruses immediately suggests host-pathogen antagonism with PKZ akin to ZBP1-E3L previously described in mammals. Indeed, the Zα_ORF112_ protein binds Z-DNA/Z-RNA in left-handed conformation resembles that of ADAR1, ZBP1, PKZ and E3L despite of low overall sequence identity and different binding kinetics (128). Significant structure resemblance between Zα_ORF112_ with Zα_PKZ_ suggests common ancestry or convergent evolution as a competitive inhibitor for PKZ sensing (24) (**Figure 2**).

In line with other ZDBD-containing proteins discussed so far, ORF112 also localizes to SGs during oxidative stress (24) (**Figure 2**).

### RNA-Binding Protein 7910 (RBP7910) of *Trypanosoma brucei*

A recent study discovered that RBP7910 from *Trypanosoma brucei* is a ZDBD-containing protein, whereby one ZDBD-like-domain was each reported at its N- and C-terminal respectively (25) (**Figure 1**). *Trypanosoma brucei* is a human-fly parasite that can cause African Trypanosomiasis or “sleeping sickness”. RBP7910 is a mitochondrial protein involved in RNA editing complexes in kinetoplastids (130). A protein sequence-based search for functional domains led to the prediction of the ZDBD-like domains, where key residues involved in Z-DNA/Z-RNA binding for Zα domain has been conserved. Nonetheless, mutational studies on these residues in RBP7910 led to lower-than-expected reduction in Z-DNA/Z-RNA-binding affinity compared to those reported for Zα_ADAR1_ (25). Further studies are needed to verify the function of ZDBD within RBP7910.

## Perspectives

### Z-DNA/Z-RNA Recognition in Antiviral Immunity

Zα_ADAR1_ is only present in the ADAR1_p150_ isoform but not in the shorter ADAR1_p110_ isoform (**Figure 1**). An alternative transcription start site is favoured when Interferon Stimulatory Response Element (ISRE) at promoter region is bound by ISGF3, thereafter catalyst for the splicing event leading to Zα-containing-ADAR1_p150_ expression (131). Similarly, other ZDBD-containing host proteins ZBP1 and PKZ are also ISGs whose expression is modulated through type I interferons (132, 133). These indicate the involvement of Z-RNA binding in the cellular regulatory events following virus infection. Furthermore, the identification of Zα domain(s) in antagonistic virus proteins reaffirms the importance of Z-RNA recognition in immune regulation (24, 116).

Fluorescence study on B-to-Z transition dynamics proposed an interesting theory on Z-DNA/Z-RNA formation (134). The study demonstrated dynamic formation of single molecule Z-DNA prior to stabilization by Zα domain, instead of induction caused by protein-nucleic acid interaction. This suggests that Zα domain function by recognizing the existing transient form of Z-DNA/Z-RNA, rather than a forced conformation change upon binding with canonical B-form dsRNA. For ADAR1_p110_, cooperative binding between three dsRBDs give rise to selectivity of A-to-I editing sites (135) (**Figure 1**). The additional Zα_ADAR1_ present only in ADAR1_p150_ can thus give rise to different editing sites during antiviral response (94) (**Figure 1**). Furthermore, alternating transition in conformation between B-form and Z-form within a dsRNA may lead to multiple editing sites on the same dsRNA, an observation described as hyperediting (136). ADAR1_p150_ is thought to effectively negate the initiation of interferon response by editing dsRNA to prevent MDA5 and PKR sensing (71, 77, 87, 137) (**Figure 2**). In contrast to ADAR1_p150_, there are two functional Zα domains in both ZBP1 and PKZ (**Figure 1**). These Zα domains are the only known nucleic acid recognition domains in respective proteins, and possibly behave synergistically for Z-DNA/Z-RNA binding. ZBP1 and PKZ function as instigators of immune response through sensing of viral nucleic acids following virus infection, *via* downstream signaling (95, 121) (**Figure 2**). The transient nature of ADAR1_p150_ binding with Z-RNA for editing activity, in contrast to the Z-RNA-dependent activation of ZBP1 and PKZ, could in part explain the difference in the Zα domains setup in these ZDBD-containing proteins. Collectively, all point towards the indispensable role of Zα domain as PAMPs/DAMPs sensor of Z-RNA, particularly in regards to antiviral defense.

### Zα-Mediated Localization to Stress Granules

Interestingly, most ZDBD-containing proteins (ADAR1_p150_, ZBP1, E3L, ORF112) have been independently reported to localize to SGs during cellular stresses (24, 90, 91, 104) (**Figure 2**). Though not explicitly verified, PKZ is also expected to localize to SGs in the same manner as ORF112, its inhibitory protein. The localization of ZDBD-containing proteins to SGs is mediated through Z-RNA binding by respective Zα domains, as mutations to key interaction residues abolished the localization pattern (91). This discovery identified a novel role of Zα domain at the forefront regulating the cellular fate and response to virus infections, and other stresses. Nevertheless, the Z-RNA substrate in SGs has not been elucidated so far owing to difficulties in complete isolation of SGs. It is also entirely plausible that favorable conditions for B-to-Z transition and Z-like steps primarily arises in the SGs, in which case the ZDBD-containing proteins is sequestered in SGs through Zα-mediated Z-RNA binding (138). Intriguingly, many proteins involved in IFN-mediated antiviral response like RIG-I, MDA5, PKR, OAS1, TRIM25 have also been found in SGs (46, 47). This sequestration may be unspecific as some are RNA binding proteins or known interaction partners. However, co-localization to SGs may enhance interactions otherwise not favorable between ZDBD-containing proteins and these antiviral sensor and effector proteins. For instance, immunoprecipitation assay revealed that PKR interacts with ADAR1 *via* dsRNA bridge, whereby the packed RNA density within SGs may give rise to additional regulatory effect between two proteins (139). In all, identifying the role of these ZDBD-containing proteins in SGs and elucidating their interaction with respective binding partners are paramount towards better understanding of antiviral innate immunity.

## Conclusion

To date, only six proteins (ADAR1_p150_, ZBP1, E3L, PKZ, ORF-112, and RBP7910) have been identified with Zα domain(s). Two common themes stood out among these proteins, where they are important in host-pathogen interaction, and they localize to SGs. The conservation of Zα domain in virus proteins is important for viral pathogenesis and immune evasion, but yet information on Z-DNA/Z-RNA motif within virus genomes is still sketchy. Similarly, while independent studies showed Z-RNA binding is responsible for protein localization to SGs, the bound substrate (Z-RNA) within SGs has not been elucidated. This is largely attributed to the transient nature when nucleic acids adapt Z-conformation that could be dependent on the cellular environment. The condensed mRNA and protein aggregates in SGs may create a favourable environment for B-to-Z transition. Research studies using nucleic acid analogues that are prone to irreversible B-to-Z transition may afford a glimpse into understanding the precise mechanism at work (140).

Nonetheless, continuous research studies on individual ZDBD-containing proteins have ascertained the central role of Zα domain and Z-DNA/Z-RNA binding in pathogen and non-self-recognition. Current studies tend to focus on ZDBD-containing proteins as a whole; instead, a reinvigorated appreciation on the key role of Zα domain in molecular innate immunity is warranted in future research. Research gaps remained; such as to understand the role of Zα-mediated localization to SGs, to address the possible redundancy between first and second Zα domains, to determine how Zα domain differentiate self from non–self-nucleic acids, and to resolve the seemingly contradictory function of ZBP1 and ADAR1_p150_ during antiviral response. Taken together, Zα-mediated nucleic acid binding represents a significant but mysterious role in immunity, and may yet offer a highly-specific Z-DNA/Z-RNA-based intervention towards immune regulation in the future.

## Author Contributions

DC, YL, and SKN conceived the framework for the manuscript. DC and SKN prepared the draft manuscript. DC, YL, and SKN revised and edited the final manuscript. All authors contributed to the article and approved the submitted version.

## Funding

This work is supported under Research University Grant for Individual (1001/CIPPT/8012264) from Universiti Sains Malaysia. DC is a recipient of Penang Future Foundation Scholarship (1709/CP039). SKN is also funded by Fundamental Research Grant Scheme (203/CIPPT/6711566) from Ministry of Higher Education, Malaysia.

## Conflict of Interest

The authors declare that the research was conducted in the absence of any commercial or financial relationships that could be construed as a potential conflict of interest.
